# Psychosocial etiology of post-traumatic stress disorder caused by war trauma among Iran–Iraq war immigrants in Mehran, Iran

**DOI:** 10.1016/j.jmh.2024.100225

**Published:** 2024-02-29

**Authors:** Ebrahim Masoudnia, Fatemeh Rahmati Farmani

**Affiliations:** Department of Sociology, Faculty of Humanity, University of Guilan, Rasht, Iran

**Keywords:** Post traumatic stress disorder, Social support, Coping strategies, Locus of Control, War-torn immigrants

## Abstract

**Background:**

Post-traumatic stress disorder (PTSD) is one of the most important and common disorders caused by war trauma. The aim of this study was to determine the relationship between psychosocial factors and PTSD in war-torn immigrants in Mehran, Iran.

**Method:**

The present study was conducted by descriptive and correlational method. The data were collected from 245 people (121 women and 124 men) from war-torn immigrants 45 years and older who migrated from Mehran to Ilam during the Iran–Iraq war. The measuring tools were as follows: Post Traumatic Stress Disorder Scale, Multidimensional Scale of Perceived Social Support (MDPSS), Coping Strategies Scale (CSS-R), Multidimensional Health Locus of Control scale.

**Results:**

The prevalence rate of PTSD among war-torn immigrants in Mehran was 35.1 %. A significant negative correlation was observed between perceived social support and PTSD (*p* < .01). Coping strategies, including seeking social support, reappraisal/adaptation, problem-focused coping, and active coping, all showed significant negative correlations with PTSD (*p* < .01). Conversely, a significant positive correlation was found between avoidance coping strategies and self-control and PTSD (*p* < .01). In addition, there was a significant positive correlation between the external locus of control (believing in chance) and PTSD (*P* < 0.01) and significant negative correlation between internal locus of control and PTSD (*P* < .01).

**Conclusion:**

Weakness in social support, locus of control and also inappropriate coping strategies against war trauma were among the strong risk factors for PTSD. Therefore, social and behavioral interventions are recommended to increase social support, teaching problem-solving skills and strengthen individual control among war-torn immigrants to reduce the risk of developing PTSD.

## Introduction

Iraq's eight-year war against Iran has had far-reaching negative effects on the mental health of the individuals and refugees directly involved. One of the most important and common disorders faced by this vulnerable population is Post Traumatic Stress Disorder (PTSD). Studies show that this disorder has many negative consequences for people who experience war trauma and leads to general dysfunction and poor quality of life (Ben-Ya'acov et al., 2004). PTSD is actually a psychological response to experiencing stress from traumatic events such as fear, helplessness, and panic (American Psychiatric Association, 2013). This disorder is often associated with depressive disorders, anxiety disorders, bipolar disorder, and substance abuse disorders (American Psychiatric Association, 2015). Symptoms of PTSD include re-experiencing a traumatic event, avoiding stimuli associated with the traumatic event, numbness of response, and increased arousal. People with PTSD also experience different forms of the aforementioned symptoms that can range from mild to very severe (Sadock et al., 2007). One of the most common symptoms of PTSD among people with war trauma is flashback or re-experiencing a traumatic event of war, which lasts a lifetime in some people. The most obvious symptoms of this disorder in soldiers with PTSD were arousal, reactivity, and negative changes in cognition and mood. In addition, the problems of these patients include the inability to communicate with others, express emotions, impulse control, aggression, guilt and the like (Modabbernia and Vaez Salehi, 1994;1989; Rahnejat et al., 2017). Thus, evidence shows that after about 30 years of the Iran-Iraq war, its traumatic effects will continue to be observed among the survivors.

The war-surviving population has a high prevalence of PTSD and depression. This statement is supported by a systematic literature search and meta-analysis of interview-based epidemiological surveys including samples from 43 war-ridden countries with a recent war history (1989–2019) (Hoppen et al., 2021). Epidemiological studies have reported varying rates of post-traumatic stress disorder related to war trauma. Studies have shown that about 30 % of soldiers returning from the Vietnam War and 17 % of soldiers returning from the Iraq War have suffered PTSD symptoms (Hoge et al., 2007; Karestan et al., 2008). In one study, the prevalence of post-traumatic stress disorder among chemical veterans was 32 % for full PTSD and 10 % for partial PTSD (Schnurr et al., 2000). Prevalence of PTSD among war-torn migrants among adult war migrants 5 years or more after displacement as of October 2014 ranges from 4.4 % to 86 %. In the case of war-torn Syrian migrants, it is reported to be 29.29 % (Bogic et al., 2015; Tinghög et al., 2017). In another study, the prevalence of PTSD due to natural disasters has been widely estimated from 8.6 % to 57.3 % (Udomratn, 2008). Different prevalence rates of PTSD have been reported in studies conducted in Iran. For example, in a study, the prevalence of post-traumatic stress disorder among chemical and non-chemical devotee was estimated at 40 % and 28.1 %, respectively (Mohaghegh-Motlagh et al., 2014). In another study, the prevalence of post-traumatic stress disorder among a number of war-torn mental patients was 44.2 (Fathi Ashtiani and Carami Nia, 2002). In studies conducted on the population that had experienced Bam earthquake trauma, after 18 months, the prevalence of PTSD was 66.7 % and, in some studies, it was 7.93 % (Eivazi et al., 2017; Noor Mohammadi and Ataei, 2009). Overall, studies show that more than half of the people who were involved in the Iran-Iraq war and more than 80 % of the veterans of this war have symptoms of PTSD.

Previous research on the etiology of post-traumatic stress disorder has focused on a range of risk factors such as psychiatric factors (e. g. childhood trauma, borderline personality disorder, paranoid traits, dependent or antisocial, genetic vulnerability to mental illness, excessive alcohol consumption). Biological factors are among other risk factors for PTSD. Some of these factors are: genetic predisposition, decreased hippocampal volume, and decreased intracranial volume, decreased corpus callosum, increased heart rate, high blood pressure, decreased cortisol levels, migraine headaches, and sleep disturbances (Sadock et al., 2007; Sadock et al., 2015).

Psychological factors such as: inadequate support system from family or friends, reactivation of unresolved psychological conflict through trauma, resuscitation of childhood trauma, and the use of defense mechanisms of denial, backlash, reversal, inability to regulate emotional states, physicalization, projection and introspection of communication with others in an aggressive and annoying manner, rupture, projective denial and guilt as defense mechanisms against underlying helplessness and perceiving an external controlling situation instead of an internal one (Sadock et al., 2007; Sadock et al., 2015). Social factors (e. g. age, gender, education, poor social and economic status, domestic and social violence, parental performance and psychological problems, lack of social and family support, and suicide) are among other predictive variables of PTSD (Sadock et al., 2007; Sadock et al., 2015).

Social support, a form of assistance typically provided by family, friends, or coworkers, plays a moderating role in the experience of traumatic events, such as war (Thoits, 2010). It can manifest in various forms: received from others (perceived support), perceived to be available when needed (perceived social support), or refers to the extent of social networks that individuals may rely on (embedded support) (Wang et al., 2021; Ling et al., 2014).

The causal relationship between perceived social support and PTSD symptoms, both before and after trauma, has been explored in diverse sample populations, encompassing veterans, patients, firefighters, survivors of sexual abuse, disaster survivors, and war survivors (Carter et al., 2016; Dworkin et al., 2018; Zalta et al., 2021). Using a cross-sectional analysis, Johnson et al. (2022) showed an inverse relationship between prior social support and later PTSD symptoms across all time points. They found that individuals who experienced trauma and had low levels of social support experienced more negative outcomes.

In a meta-analysis of 68 studies published between 1988 and 2019 and a sample of 32,402 participants, Wang et al. (2021) showed a moderate cross-sectional relationship between social support and PTSD. They found that traumatized individuals with low levels of social support fared worse.

Uchino et al. (2012) showed that people with less social support have a higher rate of mental disorders (post-traumatic stress disorder, depression, panic disorder). One study found that social support reduces the symptoms of post-traumatic stress disorder in devotees and their wives (Monson et al., 2009). Poor social support was associated with more symptoms of post-traumatic stress disorder, and perceived family social support helped to cope with illness (Morris et al., 2011). Evidence also shows that increasing social support promotes mental health, improves life quality, increases happiness and copes with stressful living conditions in post-traumatic stress disorder devotees and their wives (Hojjati et al., 2017; Eskafi Noghani et al., 2016). A study also showed that social support from friends can be effective in reducing post-traumatic stress disorder (PTSD) symptoms (Rafikhah et al., 2017).

Coping strategies have been cited as a traumatic events modulator in some studies. Coping style characterized as the contemplations or practices an individual embrace to process adversity and stress (Liu et al., 2020) and some coping styles were more adaptive, while others were more maladaptive (Lin et al., 2020). Maladaptive or emotion-focused coping styles includes denial, self-control, avoidance, reappraisal/adaptation, wishful thinking, self-harm, substance abuse, and withdrawal, while adaptive or problem-focused coping refers to natural and positive coping with problems, such as seeking social support, problem-solving with a plan, and confrontational coping (Enns et al., 2018; Brooks et al., 2020; Thompson et al., 2018).

Positive coping strategies were associated with a reduction in poor mental health outcome.

In general, problem-focused coping are associated with the reduction of poor mental health outcomes and are considered as a supportive factor for reducing anxiety and depression (Babore et al., 2020; Huang et al., 2020; Lorente et al., 2021). Using latent class analysis, Kearns et al. (2020) showed that coping based on behavioral disengagement and self-blame increased the likelihood of being in more severe PTSD–illicit substance use (e.g., cocaine) comorbidity classes. Adaptive coping strategies such as social support (Bryant-Davis et al., 2015), acceptance (Kearns et al., 2018), and cognitive reframing (Brief et al., 2011) were associated with less severe PTSD symptoms, on the contrary, maladaptive coping strategies such as avoidance (Bordieri et al., 2014), self-blame (Startup et al., 2007), and self-distraction (Kearns et al., 2018) were associated with more severe PTSD. Active coping styles modulate the relationship between symptoms of boredom and social adjustment in people with PTSD, while avoidance coping styles significantly predict PTSD symptoms (Hassija et al., 2015; Chang et al., 2003). Studies related to war have shown that the likelihood of developing PTSD in people sent to war can be predicted by observing emotion-based coping techniques and the lack of problem-focused coping strategies (Karstoft et al., 2015). In Iran, Shariati & Dehghani (2018) showed that post-traumatic stress disorder has a significant relationship with poor coping styles, emotion-focused, and symptoms of PTSD and warriors with PTSD are more likely to use emotion-based coping and immature defensive style than those without PTSD.

In summary, in the comparison of two types of coping strategies (adaptive and maladaptive), only the problem-oriented coping strategy plays a role in reducing PTSD symptoms (Zhue et al., 2021).

Another related variable that plays an important role in post-traumatic stress disorder is the locus of control (LOC). According to social learning theory, LOC is the perception of an individual concerning where the powers of control over life events are located (Rotter, 1966). The LOC has a continuum from the internal LOC to the external LOC. Internal LOC is a cognitive disposition towards understanding outcomes as a product of personal action and inactions while external locus conceptualizes outcomes as product of chance, fate, luck, and powerful others (Atilola et al., 2021). The relationship between locus of control and psychopathology has been widely investigated and the overall impression is that an individual's perception of the locus of control of life situations, affects the degree of resilience in the face of adversity. A systematic review of 29 studies whose results were published between 1997 and 2013 with a sample of 7204 people showed that greater deficit in perceived personal control is associated with higher levels of trait anxiety (Gallagher et al., 2014).

External locus of control was associated with high levels of PTSD symptoms as well as low social adjustment (Karstoft et al., 2015; Zhang et al., 2014) while an internal LOC is expected to be a protective factor against PTSD. Asberg & Renk (2014) showed that the external locus of control and low social support predicts less adaptation in people with PTSD. In relation to war trauma, research on attributions among war victims has generally shown that causal attributions and perceptions of control may be important predictors of outcomes (Larsen and Fitzgerald, 2010). In Hancock and Bryant (2018) study, it was shown that people with PTSD symptoms who believed they had no control over a series of bad events showed greater avoidance than their counterparts who felt more in control. Also, evidence also shows that the more use of internal control, the more social adjustment of PTSD devotees, and the better they can understand traumatic memories and stressful situations (Mikaeili et al., 2018).

Most studies on post-traumatic stress disorder conducted in Iran have focused on biological and psychological variables, and few studies have been conducted on the impact of psychosocial factors such as social support, coping strategies and locus of control on post-traumatic stress disorder. Therefore, the present study aimed to determine the relationship between failure in social support, coping strategies and locus of control with post-traumatic stress disorder among war migrants.

## Methods

### Procedure and participants

This research was conducted in the form of a descriptive and correlational method. The data were collected from 245 people (121 women and 124 men) of war-torn immigrants, 45 years and older, who migrated to Ilam from Mehran city during the Iran-Iraq war. They were selected using multi-stage cluster sampling method. First, the settlements of war migrants in Ilam and Mehran were identified and next, several areas were randomly selected. Then, in the selected areas, the alleys where the immigrants lived were identified and several alleys were randomly selected. All immigrants living in selected alleys aged 45 and over were then selected as a sample. Using the modified Cochran sampling formula, 245 people (121 women and 124 men) from war-torn immigrants 45 years and older were selected. Due to the lack of variance availability in the study variable (post-traumatic stress disorder) among war-torn immigrants in Mehran, a preliminary test was first performed on 30 of these immigrants. The confidence level in this study was 95 % with an erroneous estimate of 5 %.

### Inclusion and exclusion criteria

Inclusion criteria were attendance in the war conditions of Mehran during the 8-year Iran-Iraq war and being 45 years old and older. Individuals who were members of war-torn immigrant families but did not experience the conditions of war and were under the age of 45 were excluded from the study.

### Measures

#### Sociodemographic characteristics checklist

These characteristics included age, sex, marital status, level of education, income, occupation, type of housing, place of residence, history of neurological diseases, place of residence before the start of the war, age at the time of the war, being at the forefront of the war attack among war-torn immigrants.

#### Post-Traumatic stress disorder scale (PTSD)

The Foa et al. (1997) Scale was used to assess post-traumatic stress disorder. The scale consists of 17 items that were graded based on a four-point (not at all, very low, medium, high, very high) scale. The test propositions are based on the DSM-IV-TR criteria to determine the frequency and severity of each of the 17 items of PTSD on soldiers of war, victims of sexual and non-sexual assault, accidents, and a wide range of traumatic events. Foa et al. (1997) calculated the reliability coefficient of the Post-Traumatic Stress Disorder Scale using the test-retest method for the whole scale as 0.85, for the components of re-experiencing the accident as 0.92, for avoidance as 0.27, for emotional numbing as 0.90, and for hyperarousal syndrome as 0.74. The cut-off point on this scale is 50. A high score on this scale indicates the greater severity of PTSD symptoms. In the present study, the reliability coefficient using Cronbach's alpha method for the whole scale was 0.87 and for micro-scales including accident re-experience, avoidance, emotional numbness, and hyperarousal were 0.78, 0.62, 0.86, 0.74, respectively.

#### Multidimensional scale of perceived social support

The Canty-Mitchell (2000) Multidimensional Perceived Social Support Scale (MPSSS) was used to measure perceived social support. The MPSSS consists of 12 items that measure three components: perceived support from family (4 items), perceived support from significant others (4 items), and perceived support from friends (4 items). All items MPSSS were graded on a 5-point scale (strongly agree, agree, neither agree nor disagree, disagree, or strongly disagree). The scores on this scale ranged from 12 to 60. The scale constructors used the Adolescent Family Care Scale (AFCS) to assess the discriminant validity of social support subscales. They calculated the correlation between the components of family support, significant others, and social support from friend with the adolescent family care scale of (*r* = 0.76, *p* < .001), (*r* = 0.33, *p* < .001), and (*r* = 0.48, *p* < .001), respectively. They calculated the internal consistency of the Social Support Scale items using the Cronbachʼs alpha method as 0.91, 0.89 and 0.91, respectively. The MPSSS was first translated into Persian by Masoudnia (2011). In that study, three factors were identified using principal component analysis on 12 items on this scale: support from friends, support from family, and support from significant others. The internal consistency coefficients of the scale were calculated using Cronbach's alpha method as 0.78, 0.81 and 0.87, respectively, which were in a good range.

#### Modified coping strategies scale (MCSS-R)

The MCSS-R was used to measure people's coping strategies against stressful situations. The MCSS-R consists of 28 items that measure seven components with the following headings: seeking social support (4 items), active coping (2 items), reappraisal/adaptation (7 items), avoidance coping (5 items), emotion-focused coping (2 items), problem-focused coping (5 items), and self-control (2 items) (Aldwin and Revenson, 1987; Roth and Cohen, 1986). All MCSS-R scale items are graded in a 5-point scale (very high, high, medium, low, very low). This scale was first used by Masoudnia (2008) in a study on patients with rheumatoid arthritis symptoms and its validity and reliability were confirmed. In his study, the degree of reliability and internal consistency of the seven subscales of the questionnaire using Cronbachʼs alpha was in an acceptable range (seeking social support 0.90, active coping 0.86, reappraisal/adaptation 0.77, avoidance coping 0.75, emotion-focused coping 0.82, problem-focused coping 0.91).

#### Multidimensional health locus of control scale (MHLCS): short-form

The MHLCS consists of 18 items that are graded based on a five-point Likert scale (strongly agree, agree, neither agree nor disagree, disagree, and strongly disagree). The score range of the questionnaire is 18–90. A higher score on this scale indicates the external locus of control. In this study, an 8-item short form of a multidimensional health control questionnaire was used. In the multidimensional control source questionnaire, there is no specific score as the cut-off point, and the mean or middle, and more accurately Z-Score, is used for measurement. Wallston & Wallston (1978) identified three subscales by analyzing the principal components. They calculated the internal consistency of the item (Cronbach's alpha) for each component as follows: believe in chance = 0.72, powerful others = 0.78, and internal control source = 0.73. This scale was used in Iran by Masoudnia (2007). In his study, three factors were identified using principal component analysis (PCA) method and varimax rotation method on 18 items, and performing two stages of principal component analysis. These factors are: chance (external control), powerful others (external control), and internal control. Using Cronbachʼs alpha method, the internal reliability of the scale was equal to 0.89 and for the components of believe in chance, powerful others (external control) and internal control were calculated to be 0.75, 0.71 and 0.69, respectively.

### Statistical methods

Data were analyzed using SPSS software. Pearson correlation test was used to determine the relationship between social support, coping strategies and locus of control in the rate of post-traumatic stress disorder. Also, multiple hierarchical regression was used to determine the contribution of predictor variables in explaining PTSD variance.

## Results

### Description of sociodemographic characteristics of the sample

Two hundred and forty-five (121 females and 124 males) of war-torn immigrants in Mehran city were surveyed (Table 1). The average age of the participants was 54.5 with a standard deviation of 9.6. Fifty percent of the respondents were men and 49.4 % were female. In terms of marital status, 0.2 % were single, 96.7 % of the respondents were married and 1.2 percent were unanswered. In terms of education, the average level of education of the respondents was 12.62 with a standard deviation of 5.4 years. Regarding their mental health history, 19.2 % of the respondents had previously experienced a mental disorder, while 80.8 % reported no history of mental illness. In terms of age of participants during the war, the average age of respondents during the war was 16.97 with a standard deviation of 9.6. In terms of being in the war zone, 58.4 % of the respondents were in the front line, while 39.2 % of the respondents were not.

**Table 1:** Sociodemographic characteristics of the respondents.

Variable	*M*±SD	*n* (%)
Age	54.5 ± 9.6	
Gender		
Male		124 (50.6)
Female		121 (49.4)
Marital status		
single		5 (2.1)
married		237 (96.7)
Education	12.6 ± 5.4	
history of mental illness		
has a history		47 (19.2)
No history		198 (80.8)
age of participants during the war	16.9 ± 9.6	
Being in a war zone		
Yes		149 (60.8)
No		96 (39.2)
Symptoms of PTSD		
yes		86 (35.1)
no		159 (64.9)

### Social support and PTSD disorder

There was a significant negative correlation between total perceived social support and the incidence of total PTSD disorder (−0.18), psychological anesthesia and avoidance symptoms (−0.19) and hyperarousal symptoms (−0.19) (Table 2). There was also a significant negative correlation between perceived social support by friends with total PTSD (−0.15) and hyperarousal symptoms (−0.19). Perceived social support from the family was negatively related to psychological anesthesia and avoidance symptoms (−0.14). Perceived social support had a significant negative correlation with total PTSD (−0.18), psychological anesthesia and avoidance symptoms (−0.19), and hyperarousal symptoms (−0.18).

**Table 2:** Multiple correlation matrix of total social support and PTSD.

	1	2	3	4	5	6	7	8
Social support (total)	1.00							
Social support from friends	.60⁎⁎	1.00						
Social support from the family	.90⁎⁎	.31⁎⁎	1.00					
Social support from significant others	.90⁎⁎	.31⁎⁎	.78⁎⁎	1.00				
*Re*-experiencing	−0.10	−0.09	−0.06	−0.11	1.00			
Avoidance and emotional numbness	−0.19⁎⁎	−0.12	−0.14*	−0.19⁎⁎	.75⁎⁎	1.00		
hyperarousal	−0.19⁎⁎	−0.19⁎⁎	−0.12	−0.18⁎⁎	.63⁎⁎	.75⁎⁎	1.00	
PTSD total	−0.18⁎⁎	−0.15⁎⁎	−0.12	−0.18⁎⁎	.88⁎⁎	.92⁎⁎	.88⁎⁎	1.00

⁎ *p*<.05;.

⁎⁎ <0.01.

### Coping strategies and PTSD

There was a significant negative correlation between seeking social support and PTSD syndrome (−0.19), re-experiencing (−0.16), emotional numbness and avoidance (−0.16), and hyperarousal symptoms (−0.20) (Table 3). Reappraisal/adaptation had a significant negative correlation with total PTSD (−0.21), emotional numbness and avoidance (−0.24.), and hyperarousal symptoms (−0.21). Avoidance coping strategies were positively correlated with total PTSD (0.23), re-experiencing a traumatic event (0.13), emotional numbness and avoidance (0.24), and hyperarousal symptoms (0.24). Problem-focused coping had a significant negative relationship with total PTSD (−0.32), re-experiencing a traumatic event (−0.22), emotional numbness and avoidance (−0.36), and hyperarousal symptoms (−0.27). Emotion-focused coping had a significant negative correlation with emotional numbness and avoidance (−0.14). Active coping had a negative correlation with total PTSD (−0.17), re-experiencing a traumatic event (−0.13), emotional numbness and avoidance (−0.17), and hyperarousal (−0.16). Finally, the self-controlling strategy had a positive correlation with total PTSD (0.16), emotional numbness and avoidance (0.17), and a negative correlation with hyperarousal (−0.18).

**Table 3:** Correlation matrix between perceived coping strategies with total PTSD and its components.

	1	2	3	4	5	6	7	8	9	10	11
Seeking social support	1.00										
Reappraisal/adaptation	.41	1.00									
Avoidance coping	−0.03	−0.02	1.00								
Problem-focused coping	.27⁎⁎	.52⁎⁎	−0.18⁎⁎	1.00							
Emotion-focused coping	.26⁎⁎	.24⁎⁎	.09	.27⁎⁎	1.00						
Active coping	.26⁎⁎	.36⁎⁎	.04	.39⁎⁎	.33⁎⁎	1.00					
Self-controlling	.02	.13*	.24⁎⁎	.10	.07	.19⁎⁎	1.00				
Total PTSD	−0.19⁎⁎	−0.21⁎⁎	.23⁎⁎	−0.32⁎⁎	−0.10	−0.17⁎⁎	.16⁎⁎	1.00			
*Re*-experiencing a traumatic event	−0.16⁎⁎	−0.10	.13*	−0.22⁎⁎	−0.05	−0.13*	.09	.88⁎⁎	1.00		
Emotional numbness and avoidance	−0.16⁎⁎	−0.24⁎⁎	.24⁎⁎	−0.36⁎⁎	−0.14*	−0.17⁎⁎	.17⁎⁎	.92⁎⁎	.75⁎⁎	1.00	
hyperarousal	−0.20⁎⁎	−0.21⁎⁎	.24⁎⁎	−0.27⁎⁎	−0.09	−0.16⁎⁎	−18⁎⁎	.88⁎⁎	.63⁎⁎	.75⁎⁎	1.00

⁎ *p*<.05;.

⁎⁎ *p*<.01.

### Perceived controllability and PTSD

There was a significant positive correlation between belief in chance and total PTSD symptoms (0.16), re-experiencing a traumatic event (0.14), emotional numbness and avoidance (0.15), and hyperarousal (0.15) (Table 4). Also, there was a significant negative correlation between the source of internal locus of control and of total PTSD symptoms (−0.17), emotional numbness and avoidance (−0.17) and hyperarousal (−0.25).

**Table 4:** Multiple correlation matrix between perceived controllability with total PTSD and its components.

	1	2	3	4	5	6	7
belief in chance	1.00						
belief in powerful others	.34⁎⁎	1.00					
internal locus ofcontrol	−0.01	.28⁎⁎	1.00				
total PTSD	.16⁎⁎	.07	−0.17⁎⁎	1.00			
re-experiencing a traumatic event	.14⁎⁎	.11	.04	.88⁎⁎	1.00		
emotional numbness and avoidance	.15*	.07	−0.17⁎⁎	.92⁎⁎	.75⁎⁎	1.00	
hyperarousal	.15*	.03	−0.25⁎⁎	.88⁎⁎	.63⁎⁎	.75⁎⁎	1.00

⁎ *p*<.05;.

⁎⁎ *p*<.01.

### Multiple analyses

Multiple linear regression analysis was performed to determine the contribution of each variable entered in the model and was performed in two steps (Table 5). The first group of sociodemographic and clinical variables included in the first stage explained 12.4 % of the variance in PTSD. Considering the beta coefficient of the variables included in the model in the first stage, it is clear that the history of mental disorder was the only variable affecting PTSD (β=0.67;p<0.01). In the second stage, the variables of perceived social support, components of coping strategies, and perceived controllability were included in the model and explained 10.8 % of the variance in PTSD. Among the variables included in the model in the second stage, the variables of problem-focused coping (β=−.22;p<0.01), self-control (β=0.16;p<0.05), and perceived controllability (β=0.13;p<0.05) were relatively good predictors of PTSD. The hierarchical multiple regression model explained for total of 23.2 % of the variance in PTSD.

**Table 5:** Multiple hierarchical regression of PTSD.

Stage	Variable	β	T	P	$\Delta R^{2}$	Adj. $\Delta R^{2}$	F changed	P
1	Sociodemographic variables				.139	.124	9.31	.000⁎⁎
	Age	.10	1.6	.10				
	Gender	.08	.79	.42				
	history of mental illness	.67	5.1	.000⁎⁎				
	Living in a war zone	.19	1.8	.06				
2	Perceived Social Support	−0.005	−0.07	.94	.274	.232	4.56	.000⁎⁎
	Coping strategies							
	Seeking social support	−0.11	−1.7	.09				
	Reappraisal/adaptation	−0.04	−0.64	.52				
	Avoidance coping	.11	1.7	.09				
	Problem-focused coping	−0.22	−2.8	.005⁎⁎				
	Emotion-focused coping	−0.01	−0.09	.92				
	Active coping	−0.08	−1.2	.22				
	Self-controlling	.16	2.6	.01*				
	Perceived controllability	.13	2.0	.04*				

Total $R^{2}=0.274;\;R^{2}\;\text{Adj}=0.232;\;F_{\left(13,221\right)}=6.43;p<.01.$

⁎ *p*<.05;.

⁎⁎ *p*<.01.

## Discussion

The present study aimed to determine the relationship between perceived social support, coping strategies and locus of control with PTSD among war-torn migrants. The prevalence of PTSD among war-torn immigrants was 35.1 %. These results are consistent with the findings of Rahnejat et al. (2017), Fathi Ashtiani and Karaminia (2002). They also estimated the prevalence of post-traumatic stress disorder among chemical and non-chemical devotees at 40 % and 28.1 %, respectively, and at some war-torn psychiatric casualties at 44.2 %. In addition, the result of the present study is in line with the findings of Modabernia and Vaez Salehi (1986–1988), Rahnejat et al. (2017). They found that more than half of those involved in the Iraq-Iran war and 80 percent of Iranian devotees had PTSD psychological symptoms.

There was a significant negative correlation between perceived social support and PTSD among war-torn migrants. That is, the more socially supported the war victims were, the less symptoms of PTSD they had. People with perceived social support solved their problems by receiving social support from friends, relatives, and family, and as a result, perceived social support acted as a buffer against PTSD symptoms. Our results are consistent with the findings of previous studies (Johansen et al., 2022; Wang et al., 2021; Uchino et al., 2012; Morris et al., 2011; Monson et al., 2009). They found that people with less social support had higher rates of PTSD symptoms than those with higher social support. And also, perceived social support from family, friends and the like play an effective role in reducing the symptoms of post-traumatic stress in vulnerable populations, especially devotees and their wives. Moreover, our results were consistent with the Iranian findings (e. g. Hojjati et al., 2017; Eskafi Noghani et al., 2016; Rafikhah et al., 2017). They showed that increasing social support reduce the symptoms of PTSD, promote mental health, improve quality of life, increase happiness, and increase coping with stressful conditions in devotees with post-traumatic stress disorder and their wives.

It is important to note that the relationship between social support and PTSD is not only concurrent but also enduring over time. This idea has been confirmed by prospective correlational meta-analyses (Wang et al., 2021). Another point is that it is very difficult to determine whether deficits in social support caused more symptoms among Iran-Iraq war trauma survivors (social causation theory: social support - > PTSD) or, conversely, PTSD symptoms caused a decrease in social support (social selection theory: PTSD - > social support). In one hand, social causation model claims that social support buffers against PTSD, and on the other hand, the social selection model believes that individuals with PTSD, for example, would lose interest in interpersonal activities, become estranged and irritable and thus find it difficult to accept and value other's (King et al., 2006). Also, their disrupted social roles and functions would make people around them become overburdened and eventually distance them (Carter et al., 2016).

However, explaining the mechanisms that link social support to the development of PTSD is very complex. According to socio-cognitive theory (SCT) about how social support works and how to reduce post-traumatic stress, supportive social interaction can reduce negative emotions and thoughts (including negative self-esteem, significant others and negative emotions) and increase positive emotions and thoughts, especially in survivors of post-traumatic stress disorder (Baldwin, 1992). Also, SCT claims that disclosure to others of feelings and thoughts provided an opportunity for trauma assimilation and developed skills to manage negative emotions, and thus facilitated a decrease in posttraumatic distress (Wang et al., 2021). The second explanation is the theory of social cohesion about the effect of social support on post-traumatic stress disorder. According to this theory, social support causes social bonds between individuals and thus reduces psychosocial harm. Lack of social support, on the other hand, leads to despair, aimlessness, meaninglessness, depression and anxiety. Research has also shown that people who have been in contact with support groups such as family, friends, and associations have asked them for help in solving problems and gaining the skills needed to cope with stressful situations, compared to people with less social support, had a lower rate of PTSD (Sadock et al., 2015). The third explanation emphasizes the barrier and protective role of social support in dealing with stressful events. According to the direct hypothesis, social support, regardless of whether a person is under the influence of stress and psychological pressure or not, causes a person to avoid negative life experiences and has beneficial effects on health (Sarafino, 1998). Studies based on this hypothesis show that people who have higher levels of social support are less likely to suffer from mental disorders, especially post-traumatic stress disorder. Also, according to the hypothesis of indirect or shocking effect, social support is only useful and effective for people who are under stressful conditions. Under these circumstances, social support acts as a shield against the trauma of stress and plays a moderating role in the negative effects of stressful events, including war among survivors. Therefore, social support has a great impact on protecting the health of individuals and strengthening adaptive behaviors in critical situations (stress) (Cobb, 1976; Cassel, 1976).

By classifying the set of coping strategies studied into two groups of problem-focused coping strategies and emotion-focused coping strategies, our results showed that there was a significant negative correlation between PTSD and the components of seeking social support, problem-focused coping, active coping, and reappraisal/adaptation among war-torn migrants. War-torn individuals used mechanisms such as family and social support, positive thinking, problem-solving, positive daily activities, and healthy recreation to solve their problems were less likely to develop PTSD. These results were consistent with previous findings (Babore et al., 2020; Huang et al., 2020; Lorente et al., 2021; Kearns et al., 2020; Hassija et al., 2015; Ozer et al., 2003; Slanbekova et al., 2017; Ahmadizadeh et al., 2012; RahNejat et al., 2014). In general, these studies showed that the use of problem-focused coping strategies such as seeking social support, problem-solving, and active coping play a mediating role in the relationship between PTSD and the severity of psychological symptoms, and are effective in increasing social adjustment in people with PTSD. Also, using a reappraisal/adaptation strategy modifies negative thoughts and reduces the intensity of arousal in patients with PTSD. On the other hand, the results of our study showed that there was a significant positive correlation between PTSD and avoidance coping strategies and self-control among war-torn migrants. War-torn people who are frustrated and indifferent to problems and tend to engage in negative behaviors such as smoking, sedation, isolation, and avoidance of thinking in the face of stressful situations are more likely to develop PTSD. This result is consistent with the findings of Karstoft et al. (2015), Chang et al. (2003), Shakeri and Sadeghi (2003), Shariati and Dehghani (2016).

Coping styles such as avoidance, emotion-focused coping, and self-control coping were significantly associated with PTSD symptoms, and that PTSD warriors were more likely to use emotion-focused coping styles. Instead of dealing with stress decisively, these people try to forget the stressor by doing things like extreme entertainment, misuse of drugs, alcohol, and so on. Generally, comparing problem and emotion-focused coping strategies, only problem-focused coping was effective in reducing PTSD symptoms (Page et al., 2021). Our results are not consistent with the findings of some studies. For example, Xu et al. (2023) found that behavioral disengagement coping, such as giving up the attempt to cope, was negatively related to psychological distress but was positively related to depression and insomnia. Also, they found that active coping was positively associated with symptoms of anxiety, and planning was positively associated with symptoms of insomnia.

According to the theory of Cutrona and Russell (1990), Folkman and Lazarus, 1980, Lazarus and Folkman, 1984, stress leads to illness or negative outcomes when people evaluate situations negatively and do not use appropriate and effective coping responses, such as problem solving and emotion control. To prevent such a situation, this theory suggests strengthening more adaptive assessments and more effective coping. In fact, social support improves assessments and coping with stress and also it matches the demands of stressful stimuli with available resources. Cognitively, how individuals evaluate situations (challenge, threat, profit, loss) is the first determining factor in dealing with them. All of these assessments are influenced by environmental demands as well as individual beliefs, values, and commitments. Based on this theory, mature, rational and balanced individuals are more likely to resort to problem-focused strategies in dealing with stressful situations such as attracting social and family support, realism, attention to the positive points of the problem. In contrast, sensitive, immature, and psychologically unbalanced individuals often use emotion-focused coping strategies (anger, isolationism, violence) in dealing with stressors.

Studies have shown that veterans with recurrent post-traumatic stress disorder are more likely to use the avoidance method, which describes methods to avoid the problem. In such a way that the person affected by stress creates a tragedy and denies its existence due to the lack of mental overcoming of stress. Instead of dealing with stress decisively, he/she tries to forget the stressor by doing things like extreme entertainment, misuse of drugs, alcohol, etc. (Shakery and Sadeghy, 2003). In another study, it was shown that the use of reassessment strategy corrects negative thoughts and reduces the intensity of arousal in patients suffering PTSD (Rahnejat et al., 2014).

By dividing the control source into two categories of internal and external, our results showed a significant negative and positive correlation in terms of PTSD, respectively. War-torn people with the source of internal control believed that with self-control, self-esteem, and self-confidence, they could overcome their problem and disease. As a result, reducing the stress of war trauma led to a reduction in the PTSD symptoms. Our results confirm some of the findings of previous studies (Hancock and Bryant, 2018; Zhang et al., 2014; Karstoft et al., 2015; Larsen et al., 2010). War-torn people with an external locus of control (believing in chance) believed that their health depended on luck or considered themselves prone to disease, were more likely to develop PTSD. This result was consistent with the findings of Karstoft et al. (2015) and Asberg & Renk (2014). They also showed that traumatic events, including war, due to their unpredictable and uncontrollable nature, can cause or exacerbate the symptoms of PTSD in their victims. The source of external control as well as low social support predicts low social adjustment in people with PTSD. According to social learning theory, self-control is seen as potential forces and talents to respond to different social situations. Some people with the personality trait of the source of internal control, consider themselves responsible for their failures or successes, and the reinforcement they receive is under the control of their own behaviors and characteristics. While individuals with the personality trait of the source of external control, attribute their performance to the environment, luck, fortune and others. These people believe that their behaviors and abilities have no effect on the reinforcements they receive. They often do not attempt to improve their situation. As a result, it can be stated that people who have complete control over their lives and have characteristics such as self-esteem, self-confidence, and self-fulfillment, compared to those who have a source of external control (belief in luck, destiny, etc.) are less likely to suffer from post-traumatic stress disorder. They can also better understand and adapt to traumatic memories and stressful situations (Rutter and Hochreich,1975; Schultz and Schultz, 2016). Individuals with PTSD symptoms who hold negative beliefs about personal control, including appraising symptoms as uncontrollable or having poor self-efficacy, show poorer outcomes following trauma exposure and treatment (Ayers et al., 2007).

## Limitations and future research

Due to some limitations, it is necessary to generalize the findings of this study with caution. The first limitation of the present study relates to the tool for measuring variables. Since the measurement of variables such as perceived social support, coping strategies, and locus of control is done with the help of indirect and self-assessment tools, the completion of these questionnaires is greatly influenced by people's interests in self-introduction, ostentation and perception management strategies (Farnham et al., 1999). Thus, as Joinson (1999) has shown, when people are in anonymous conditions, their scores on these scales differ compared to familiar ones. Therefore, it is suggested to future researchers that in addition to using indirect tools, they use more direct tools that are less exposed to subjective and memory-based judgments. Therefore, it is suggested to future researchers that in addition to using indirect tools, they use more direct tools that are less exposed to subjective and memory-based judgments. The second limitation of this study is related to the participants of this study. Because it was very difficult to reach people with eight years of war experience, the present study limited the number of samples. Therefore, it is suggested to future researchers to focus their study on wider populations.

## CRediT authorship contribution statement

**Ebrahim Masoudnia:** Writing – review & editing, Writing – original draft, Visualization, Validation, Supervision, Software, Resources, Project administration, Methodology, Investigation, Formal analysis, Data curation, Conceptualization. **Fatemeh Rahmati Farmani:** Resources, Data curation, Conceptualization.

## Declaration of competing interest

All authors declare that they have no conflict of interest.
